# MiR‐22 modulates the expression of lipogenesis‐related genes and promotes hepatic steatosis *in vitro*

**DOI:** 10.1002/2211-5463.13026

**Published:** 2020-11-27

**Authors:** Zhuo Yang, Wen Qin, Junsheng Huo, Qin Zhuo, Jingbo Wang, Liyuan Wang

**Affiliations:** ^1^ National Institute for Nutrition and Health Chinese Center for Disease Control and Prevention Key Laboratory of Trace Element and Nutrition National Health Commission of China Beijing China

**Keywords:** lipid metabolism, miR‐22, nonalcoholic fatty liver disease, obesity, Sirt1

## Abstract

Nonalcoholic fatty liver disease (NAFLD) is highly correlated with obesity, and lifestyle changes to reduce weight remain the main therapeutic approach. The noncoding RNA miR‐22 has previously been reported to be highly abundant in the sera of NAFLD patients. In addition, miR‐22 directly targets peroxisome proliferative‐activated receptor, Pgc‐1α, peroxisome proliferator‐activated receptor α, and sirtuin 1 (Sirt1), which are important factors involved in fatty acid metabolism. Given that miR‐22 directly targets genes involved in the control of metabolism and obesity, we investigated whether miR‐22 contributes to metabolic alterations induced by obesity. We observed increased expression of miR‐22, decreased expression of *Sirt1*, and alterations in the expression of adipogenesis‐related genes in a mouse model of obesity and a human hepatocyte cell line. We identified that miR‐22 and the 3′‐UTR of *Sirt1* are complementary. Mutation of the complementary fragment abolishes the ability of miR‐22 to regulate the *Sirt1* gene. Furthermore, treatment of hepatic steatosis cells with miR‐22 mimics or inhibitors showed that miR‐22 can promote hepatic steatosis, and miR‐22 inhibitors effectively reduced triglyceride levels without affecting cell activity. Finally, we validated that miR‐22 has similar effects on downstream lipid metabolism‐related genes. Our data reveal the pathways and mechanisms through which miR‐22 regulates lipid metabolism and suggest that miR‐22 inhibitors may have potential as candidate drugs for NAFLD and obesity.

## Graphical abstract

We observed that miR‐22 can promote hepatic steatosis. Conversely, steatosis was alleviated after inhibition of miR‐22, indicating that miR‐22 is involved in fat deposition. Furthermore, miR‐22 was validated to have effects on downstream lipid metabolism‐related genes, revealing the pathways through which miR‐22 regulates lipid metabolism. Therefore, miR‐22 may be a promising target for the treatment of NAFLD and obesity.

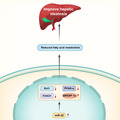

**Abbreviations**
FFA: free fatty acids
FOXO1: forkhead box protein O1
GAPDH: glyceraldehyde‐3‐phosphate dehydrogenase
HFD: high‐fat diet
miRNAs: microRNAs
NAFLD: nonalcoholic fatty liver disease
NAS: NAFLD activity score
NASH: nonalcoholic steatohepatitis
PPARα: peroxisome proliferator‐activated receptor α
SCD: standard chow diet
Sirt1: sirtuin 1
SREBP‐1c: sterol regulatory element‐binding protein‐1c
TG: triglyceride

Nonalcoholic fatty liver disease (NAFLD) is an obesity‐related metabolic complication. In severe cases, NAFLD can cause hepatocellular carcinoma or progress to cirrhosis, requiring liver transplantation [1, 2]. NAFLD is characterized by inappropriate ectopic lipid deposition in which microRNAs (miRNAs) play a pivotal role [3].

Lipid homeostasis is precisely controlled by complex genetic and metabolic networks in the liver. In recent years, many pieces of evidence suggested that miRNAs are related to lipid metabolic disorders of the liver. MiR‐122 is the most abundant miRNA in the human liver and was the first miRNA to be identified as playing a crucial role in lipid metabolism. Some researchers found that miR‐122 is involved in lipid synthesis, catabolism, and secretion. Moreover, miR‐122 has antitumor functions in the liver and was found to regulate the cholesterol levels in human plasma [4, 5, 6, 7]. In a study of NAFLD, Cheung *et al*. found that miR‐27b acts as a regulatory hub of hepatic lipid metabolism [8]. In addition, miR‐34a has been shown to contribute to hepatic steatosis by inhibiting sirtuin 1 (Sirt1) [9]. Soh *et al*. [10] found that miR‐30c could reduce lipid biosynthesis and lipoprotein levels, and increasing miR‐30c levels was found to be helpful for treating hyperlipidemia and other disorders. MiR‐33a and miR‐33b are key regulators of cholesterol homeostasis in both mice and nonhuman primates [11, 12]. Recently, mir‐223 has been recognized as a major coordinating factor of cholesterol metabolism [13]. Stacchiotti *et al*. [14] found that the beneficial effect of melatonin on NAFLD/nonalcoholic steatohepatitis (NASH) in mice was associated with reduced miR‐34a‐5p levels in the presence of Sirt1. These studies revealed that hepatic miRNAs are at the core of lipid homeostasis regulation, with key roles in NAFLD and other liver diseases.

The functions of miR‐22 in cells are various, and the biological effects of miR‐22 on aging, energy supply, angiogenesis, cell proliferation, migration, invasion, metastasis, and apoptosis have been extensively studied [15, 16, 17]. In various cancers, the inhibition or promotion of miR‐22 was found to be greatly amplified by the formation of positive or negative feedback loops related to miR‐22. MiR‐22 and numerous related molecules constitute a complex signaling network with miR‐22 as the core. López‐Riera *et al*. [18] found that some miRNAs, including miR‐22, were highly abundant in the sera of NAFLD patients. In addition, miR‐22 directly targets peroxisome proliferative‐activated receptor, Pgc‐1α, peroxisome proliferator‐activated receptor α (PPARα), and Sirt1, which are important factors involved in fatty acid metabolism [19]. Given that miR‐22 directly targets genes such as Pgc‐1α, PPARα, and Sirt1, which are involved in the control of metabolism and obesity, it would be highly valuable to investigate whether miR‐22 may contribute to metabolic alterations induced by obesity.

In this study, miR‐22 levels were shown to be significantly increased in obese model mice, but the protein expression of Sirt1 was decreased. In order to clarify the function of miR‐22 in lipid metabolism, we treated hepatocytes with free fatty acids (FFA) *in vitro* and found that the levels of miR‐22 increased during adipogenesis. To further understand how miR‐22 regulates lipid metabolism, we examined its influence on the Sirt1 signaling pathway, lipid‐related genes, and pro‐inflammatory cytokines.

## Materials and methods

### Cell lines and treatment conditions

Human normal hepatocyte cell line L02 was obtained from the Chinese Center for Disease Control and Prevention, and cultured in RPMI 1640 medium supplemented with 10% (v/v) FBS at 37 °C in a humidified atmosphere comprising 5% CO_2_. To induce fat accumulation, the cells were treated with 0.5 mm FFAs (oleate: palmitate = 2 : 1, 10% BSA) in the absence of FBS.

### Mouse model of obesity

Male C57BL/6 mice (4–6 weeks, 18–20 g) were obtained from SPF Biotechnology (Beijing, China). All mice were housed in pathogen‐free facilities and maintained at 25 °C and a 12‐h day/night cycle, with food and water available *ad libitum*. The mice were randomized (*n* = 10 in each group) to the standard chow diet (SCD) or the high‐fat diet (HFD) (75% SCD, 2% cholesterol, 15% lard, and 8% yolk powder) for 12 weeks to establish the obesity mouse model. Metabolic phenotypes and serum parameters were analyzed. The livers were quickly excised, cleaned, and preserved in liquid nitrogen until further use. All animal experiments were approved by the Ethics Committee of the National Institute for Nutrition and Health, Chinese Center for Disease Control and Prevention, Beijing, China.

### Cell transfection

The miR‐22 mimic/inhibitor and the negative control miRNA‐NC were synthesized by RiboBio (Guangzhou, China). Cells were transfected with the miRNAs and plasmids using Lipofectamine 2000 reagent (Invitrogen, Carlsbad, CA, USA). The transfected cells were treated with FFA and harvested after 48 h.

### Dual‐luciferase assay

The pMIR vector (Ambion, Austin, TX, USA) was used to express luciferase and as the template vector for 3′‐UTR reporter assays. Wild‐type and mutant 3′‐UTRs from Sirt1 mRNA were cloned and inserted into the 3′‐UTR of pMIR. Then, L02 cells were transfected with pMIR and miR‐22 mimic using Lipofectamine 2000, and pRL‐CMV was used as an internal reference. The cells were lysed for the analysis of relative luciferase activity after 36 h post‐transfection. The activities of firefly and *Renilla* luciferases were measured using the dual‐luciferase reporter assay system kit (Promega, Madison, WI, USA) according to the instructions. The relative luciferase activities were calculated as firefly luciferase (pMIR) divided by *Renilla* luciferase (pRL‐CMV).

### Real‐time PCR

Total RNA was extracted using TRIzol (Invitrogen) and transcribed into cDNA using the PrimeScript RT Master Mix Kit (TaKaRa, Dalian, China). Real‐time quantitative PCR was conducted using the SYBR Green Master Mix (TaKaRa) on a LightCycler 480 system (Roche, Basel, Switzerland). The temperature program encompassed initial denaturation at 94 °C for 30 s, followed by 40 cycles of 94 °C for 5 s and 60 °C for 10 s. The primers used for the quantification of Sirt1, PPARa, forkhead box protein O1 (FOXO1), sterol regulatory element‐binding protein‐1c (SREBP‐1c), and glyceraldehyde‐3‐phosphate dehydrogenase (GAPDH) are listed in Table 1. The miR‐22 qRT‐PCR primers were designed and synthesized by RiboBio. The average C*_t_* values were normalized to GAPDH, and the miRNA expression levels were normalized to U6. The $2^{‐\Delta \Delta C_{t}}$ method was used to calculate the fold change between the experimental groups and control. All reactions were carried out in triplicates.

**Table 1:** Primers used for qRT‐PCR analysis.

Gene	Primer sequences (5′→ 3′)
hSIRT1	Forward primer: TGTGTCATAGGTTAGGTGGTGA Reverse primer: AGCCAATTCTTTTTGTGTTCGTG
mSIRT1	Forward primer: GACGCTGTGGCAGATTGTTA Reverse primer: GGAATCCCACAGGAGACAGA
hGAPDH	Forward primer: ACAGTCAGCCGCATCTTCTT Reverse primer: GTTAAAAGCAGCCCTGGTGA
mGAPDH	Forward primer: AGGTCGGTGTGAACGGATTTG Reverse primer: TGTAGACCATGTAGTTGAGGTCA
hPPARa	Forward primer: TTCGCAATCCATCGGCGAG Reverse primer: CCACAGGATAAGTCACCGAGG
hFOXO1	Forward primer: TCGTCATAATCTGTCCCTACACA Reverse primer: CGGCTTCGGCTCTTAGCAAA
hSREBP‐1c	Forward primer: CCTGGCCTATTTGACCCACC Reverse primer: TTCAATGGAGTGGGTGCAGG

### Western blotting

Protein lysates were electrophoretically separated by SDS/PAGE and transferred to polyvinylidene fluoride membranes. Immunoblotting was performed with the corresponding primary antibody (anti‐Sirt1 antibody at 1 : 1000, anti‐PPARa antibody at 1 : 1000, anti‐FOXO1 antibody at 1 : 2000, anti‐SREBP‐1c antibody at 1 : 1000, and anti‐GAPDH antibody at 1 : 1000; Cell Signaling Technology, Danvers, MA, USA) for 1 h at 37 °C. The signals were visualized using an HRP‐conjugated secondary antibody (1 : 5000 dilution; Cell Signaling Technology) with a chemiluminescence detection system (Amersham Bioscience, Piscataway, NJ, USA).

### Histological analysis

Hematoxylin–eosin staining was done according to the standard protocol to examine liver histomorphology. Liver tissue was removed from mice and fixed in formalin and then embedded in paraffin. Paraffin sections (2–5 µm) were stained with hematoxylin and eosin. Histopathological identification was performed by researchers blinded to the treatments. The histological features were assessed using the NASH clinical research network scoring system. The NAFLD activity score (NAS) was calculated as the unweighted sum of the scores for steatosis (0–3), lobular inflammation (0–3), and ballooning (0–2).

### Measurement of triglyceride levels

The cells were homogenized and extracted with absolute ethyl alcohol. Then, the supernatant of lysed cells which were collected by centrifugation at 2000 ***g*** for 10 min or plasma of mice was used for subsequent triglyceride (TG) quantification using an enzymatic assay kit (Applygen, Beijing, China) following the manufacturer's instructions. The reaction mixture was incubated for 15 min, after which the absorbance at 510 nm was measured. The experiments were repeated three times.

### Oil red O staining

Oil Red O staining was used to evaluate lipid droplets in L02 cells and liver tissues. The cells were fixed in 10% formalin for 10 min, rinsed in isopropanol, and incubated with Oil Red O reagent for 0.5 h, followed by hematoxylin counter‐staining for 1 min. The stained cells were observed under a fluorescence microscope. The mouse liver tissues were first fixed in 4% paraformaldehyde and then embedded in OCT and sectioned into slices of 2–5 μm thickness using a cryotome. These sections were then dewaxed in xylene and rehydrated. After rinsing with PBS, the tissue sections were incubated in oil red O solution for 30 min, followed by hematoxylin counter‐staining for 1 min. After washing and dehydration, the oil red O and hematoxylin‐stained sections were mounted for fluorescence microscopy imaging. The experiments were repeated at least three times.

### Cell viability assay

Cell survival was examined using the CCK‐8 assay kit (Dojindo, Kumamoto, Japan). Cells were seeded at the density of 5 × 10^3^ and incubated overnight. Subsequently, 10 μL of the CCK‐8 reagent was added and incubated for 5 h at 37 °C, after which the absorbance was measured at 450 nm. The experiments were repeated three times.

### Assay for pro‐inflammatory cytokines

L02 cells were seeded and transfected as described above. After 48 h, the levels of the pro‐inflammatory cytokines TNF‐α and IL‐6 in the culture were measured using corresponding enzyme‐linked immunosorbent assay kits (Abcam, Cambridge, UK). The absorbance of the samples at 450 nm was measured, and the determination was repeated three times.

### Statistical analysis

Statistical analysis was performed using spss 12.0 (IBM Corp., Armonk, NY, USA). The data are presented as averages of at least triplicate biological replicates ± standard deviation. The differences between groups were assessed using Student's *t*‐test, and a *P*‐value < 0.05 was considered to indicate a statistically significant difference.

## Results

### Changed miR‐22 levels and Sirt1 expression in the mouse model of obesity

**Fig. 1:**
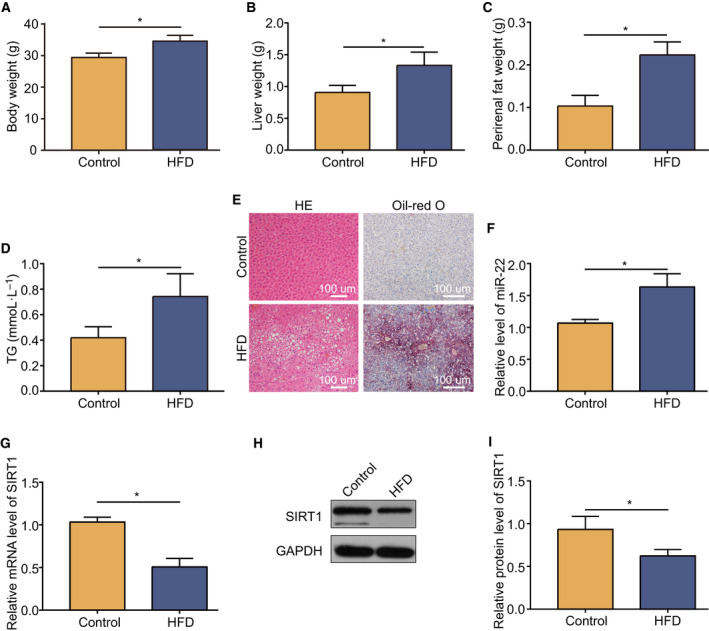
Upregulation of miR‐22 and downregulation of Sirt1 in the obesity mouse model. Weight changes of the body (A), liver (B), and perirenal fat (C) in the NAFLD model. (D) TG concentration in the obesity model. (E) HE and Oil Red O stained liver tissues at 100× magnification. Scale bars: 100 µm. The level of miR‐22 (F) and Sirt1 mRNA (G) in the obesity mouse model compared with control tissues examined by quantitative real‐time PCR. (H) Western blot analysis of Sirt1 protein expression in the obesity model and control. (I) Downregulation of Sirt1 protein expression in the obesity model. Data were presented as mean ± SD for three independent experiments. Student’s *t*‐test was conducted. ^*^ *P* < 0.05.

Firstly, we established an obesity model in mice induced by a HFD, which was demonstrated by the significantly increased body and liver weight (Fig. 1A,B). Moreover, the perirenal fat weight and the level of TG increased by 120.0% and 71.4%, respectively (Fig. 1C,D). HE staining of liver tissues showed that there were more dispersed lipid vacuoles in HFD mice than in control mice (Fig. 1E). Histological scoring of NAFLD revealed that the obesity model mice fed the HFD displayed signs of NAFLD (Table 2). Accordingly, oil red O staining revealed remarkable deposition of lipid droplets in the livers of HFD mice (Fig. 1E), which proved that the obesity model was established successfully. According to quantitative real‐time PCR analysis, the relative miR‐22 levels were increased by 53.0% in the obese model mice (Fig. 1F). By contrast, the RNA levels of Sirt1 were evidently reduced by 50.5% (Fig. 1G). Further western blot analysis confirmed the reduced protein levels of Sirt1 (Fig. 1H,I).

**Table 2:** NAS in SCD and HFD mice. The total NAS score is the sum of the values recorded for each category: 1–2 = no NASH; 3–4 = borderline; 5–8 = NASH.

	SCD (*n* = 6)	HFD (*n* = 6)
Steatosis	0.0 ± 0.0	2.5 ± 0.5*
Inflammation	0.2 ± 0.2	0.5 ± 0.5
Ballooning	0.0 ± 0.0	0.0 ± 0.0
NAS	0.2 ± 0.2	3.0 ± 0.5*

* *P* < 0.05 versus control.

### Free fatty acids induced miR‐22 upregulation and lipogenesis in human hepatocytes

**Fig. 2:**
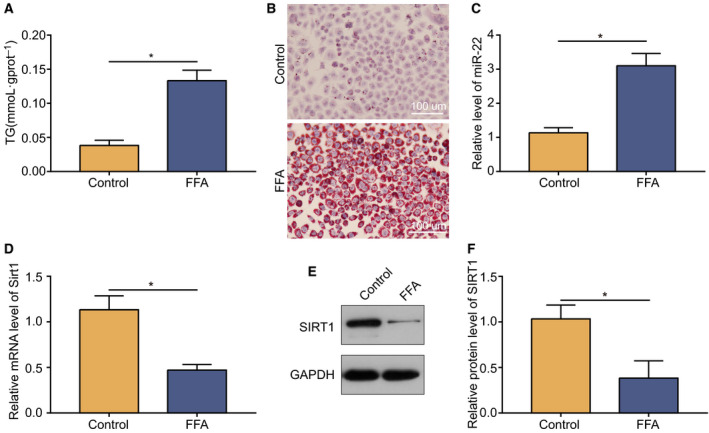
*In vitro* validation of increased levels of miR‐22 and downregulation of Sirt1 expression in human hepatocytes. (A) TG concentration in FFA‐induced hepatocytes versus the control group. (B) Oil Red O staining for lipid droplets in hepatocytes. Scale bars: 100 µm. (C) MiR‐22 level in FFA‐induced hepatocytes versus control. (D) mRNA level of Sirt1 in FFA‐induced hepatocytes versus control. (E) Immunoblotting of protein Sirt1 in FFA‐induced hepatocytes and control. (F) Downregulation of Sirt1 protein expression in FFA‐induced hepatocytes. Data were presented as mean ± SD for three independent experiments. Student’s *t*‐test was conducted. Data were presented as mean ± SD for three independent experiments. Student’s *t*‐test was conducted. ^*^ *P* < 0.05.

To verify the above results, we treated the human hepatocyte line L02 with FFAs. As shown in Fig. 2A,B, the TG levels in the FFA‐induced hepatocytes were tripled, and obvious lipid accumulation was observed in the FFA‐induced cells. Similarly, miR‐22 expression was significantly higher than in the control (Fig. 2C). Furthermore, Sirt1 expression decreased significantly at both the mRNA and protein levels (Fig. 2D‐F). Compared with the results in 3.1, the changes of miR‐22 levels and Sirt1 expression in human hepatocytes were much higher than in the obesity mouse model.

### miR‐22 suppresses the expression of Sirt1 by targeting its 3’‐UTR

**Fig. 3:**
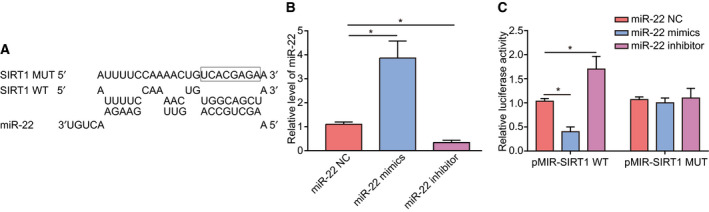
SIRT1 was a direct target gene of miR‐22. (A) The sequence of miR‐22, 3′‐UTR of Sirt1 gene and Sirt1 mutation. (B) The influence of miR‐22 mimics/inhibitor on the level of miR‐22. (C) The binding site was confirmed via a luciferase assay. Data were presented as mean ± SD for three independent experiments. Student's *t*‐test was conducted. ^*^ *P* < 0.05.

Sequence alignment revealed that miR‐22 and the 3′‐UTR of Sirt1 are partially matched (Fig. 3A). Therefore, we predicted that the 5′‐UGGCAGCU‐3′ sequence is the direct target of miR‐22. To identify the relationship between them, we constructed a Sirt1 mutation containing the sequence 5′‐UCACGAGA‐3′ instead of 5′‐UGGCAGCU‐3′ (Framed in Fig. 3A). Additionally, L02 cells were transfected with miR‐22 mimic/inhibitors to change the expression level of miR‐22 (Fig. 3B). Luciferase expression analysis showed that the mutant sequence lost its response to miR‐22, confirming the target site (Fig. 3C).

### Effects of the miR‐22 mimics/inhibitor on steatosis in hepatocytes *in vitro*

**Fig. 4:**
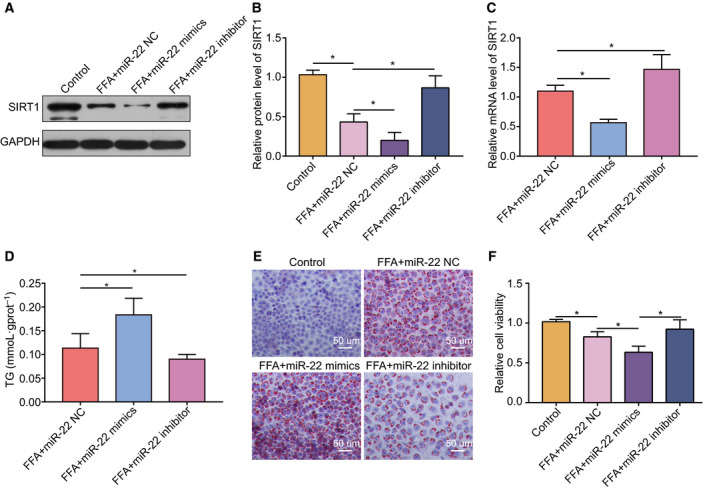
Effect of miR‐22 mimics/inhibitor on lipid accumulation in human hepatocytes. (A)/(B) Effect of miR‐22 mimics/inhibitor on Sirt1 protein levels in hepatocytes. (C) Effect of miR‐22 mimics/inhibitor on Sirt1 mRNA levels in hepatocytes. (D) Effect of miR‐22 mimics/inhibitor on TG levels in hepatocytes. (E) Effect of miR‐22 mimics/inhibitor on the lipid content of hepatocytes examined via oil red O staining and microscopy. Scale bars: 50 µm. (F) Effect of miR‐22 mimics/inhibitor on relative cell viability of hepatocytes. Data were presented as mean ± SD for three independent experiments. Student's *t*‐test was conducted. ^*^ *P* < 0.05.

To evaluate the influence of miR‐22 on FFA‐induced hepatic steatosis *in vitro*, we used miR‐22 mimic and inhibitor to treat hepatocytes. Cells that had been treated with 100 nmol miR‐22 NC, miR‐22 mimics, or miR‐22 inhibitor were induced with 0.5 mm FFAs. The results showed that the protein and mRNA expression of Sirt1 was significantly decreased in cells treated with miR‐22 mimic compared to the control, while the miR‐22 inhibitor raised the expression levels of Sirt1 (Fig. 4A‐C). The TG levels in the miR‐22 mimics transfected hepatocytes were increased and the miR‐22 inhibitor did the opposite (Fig. 4D). Staining with oil red O to visualize lipid droplets showed that a significant increase of the cellular lipid content after FFA treatment compared with normal cells. However, the effect of transfection with mimics and inhibitor on FFA‐treated cells showed significant differences (Fig. 4E). While the miR‐22 mimics enhanced the effect of FFA treatment, resulting in excessive lipid accumulation, the inhibitor attenuated the effect of FFA, resulting in a significant reduction of the cellular lipid content. In addition, we examined the relative cell viability and found that FFA + miR‐22 mimics had the greatest effect, followed by FFA, while the viability of cells treated with FFA + miR‐22 inhibitor was closest to that of control cells(Fig. 4F).

### MiR‐22 modulates the expression of lipogenesis‐related genes

**Fig. 5:**
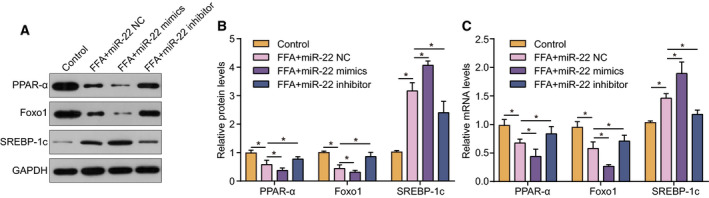
The expression of lipogenesis‐related genes was regulated by miR‐22. (A) and (B) The effect of miR‐22 mimics/miR‐22 inhibitor on the expression of lipogenesis‐related proteins targeted by Sirt1. (C) The influence of miR‐22 mimics and inhibitor on lipogenesis‐related mRNAs targeted by Sirt1. Data were presented as mean ± SD for three independent experiments. Student's *t*‐test was conducted. ^*^ *P* < 0.05.

To clarify the mechanism by which miR‐22 promotes lipid accumulation in hepatocytes, we examined the effects of miR‐22 on lipogenic pathways. We found that the protein expression of PPAR‐α and FOXO1 was significantly decreased in cells treated with miR‐22 mimic compared to the control, while the miR‐22 inhibitor raised the expression levels of PPAR‐α and FOXO1 (Fig. 5A,B). By contrast, the protein expression of SREBP‐1c was positively correlated with miR‐22 and was consistent with the induction effect of FFAs. These data suggested that miR‐22 may affect a series of principal proteins in lipid metabolism *in vitro*. The mRNA levels of PPAR‐α, FOXO1, and SREBP‐1c showed similar trends with the above results (Fig. 5C).

### MiR‐22 regulates the expression of pro‐inflammatory cytokines in hepatocytes

**Fig. 6:**
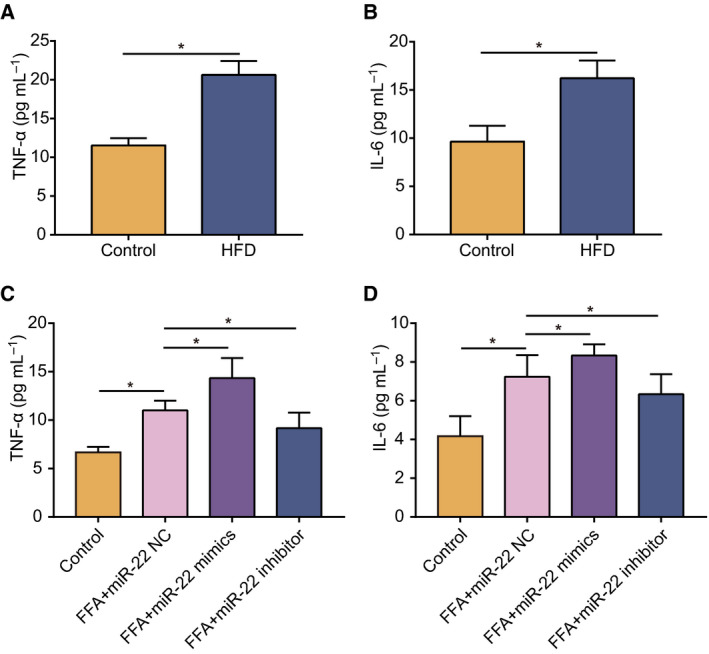
MiR‐22 regulates the expression of pro‐inflammatory cytokines in hepatocytes. (A) The expression levels of TNF‐α in the obesity model mice fed a HFD and control mice. (B) The expression levels of IL‐6 in the obesity model mice and controls. (C) The effect of miR‐22 mimics/miR‐22 inhibitor on TNF‐α in the steatotic hepatocyte model. (D) The effect of miR‐22 mimic and inhibitor on IL‐6 in the steatotic hepatocyte model. Data were presented as mean ± SD for three independent experiments. Student's *t*‐test was conducted. ^*^ *P* < 0.05.

Inflammation usually occurs in steatotic models, and changes in the levels of the pro‐inflammatory factors TNF‐α and IL‐6 are widely used to indicate the condition of inflammation. By detecting the obese mouse model established above, we found that the level of TNF‐α and IL‐6 increased significantly (Fig. 6A,B), indicating that the inflammatory state of mice was activated. Afterward, the results in steatotic cells produced by FFA treatment showed that the levels of TNF‐α and IL‐6, respectively, increased by 65.0% and 73.6%. In addition, miR‐22 mimics further aggravated inflammation, while miR‐22 inhibitor relieved inflammation (Fig. 6C,D). Therefore, miR‐22 can regulate inflammatory effects of steatosis by affecting the expression of the pro‐inflammatory factors TNF‐α and IL‐6.

## Discussion

Abnormal fat metabolism can cause obesity and NAFLD, which in turn interferes with normal metabolism throughout the body. As the most common liver disease worldwide, NAFLD affects human health and causes an enormous socioeconomic burden. This study revealed that miR‐22 expression was increased in obese model mice *in vivo* and in human hepatocytes *in vitro*, while Sirt1 was downregulated. Subsequently, the Sirt1 mRNA was verified to contain a direct binding site of miR‐22 via the dual‐luciferase reporting system. Afterward, it was found that transfection with an inhibitor of miR‐22 effectively alleviated lipid accumulation, which may be due to the activation of lipid metabolism‐related signaling pathways. Therefore, these findings deepen the understanding of the pathogenesis of NAFLD and the regulatory pathway of miR‐22.

There are many studies on gene regulation by miR‐22. Xu *et al*. reported that as a novel aging‐related miRNA, overexpression of miR‐22 induces cancer cell senescence and can also reduce cell viability to play a cancer suppressor role. MiR‐22 knockdown in presenescent fibroblasts reduced the cell size and made the cells more compact [20]. We also found that miR‐22 could reduce the viability of hepatocytes, but the cell size did not change significantly, which may be due to functional differences of miR‐22 in different tissues. Furthermore, we found that miR‐22 inhibitors could reduce the fat content of hepatocytes without affecting cell viability. MiR‐22 has also been found to target many genes encoding cancer‐associated proteins, including the TET2 tumor suppressor, HDAC6, ERBB3, CDC25C, EVI‐1, and P21 [21, 22, 23, 24], which play different roles in different types of cancer. In addition, miR‐22 is also an important regulator of cardiac remodeling, associated with a hypertrophic response, sarcomere reorganization and altered metabolic program [25]. Diniz *et al*. found that loss of miR‐22 prevented dyslipidemia induced by a HFD, but did not affect weight gain, insulin resistance, or cardiac hypertrophy [26].

Sirt1 is a protein deacetylase belonging to the sirtuin family. It can deacetylate substrates (many transcription factors and cofactors) which may lead to their activation or inactivation, thus affecting downstream genes. Initially, Sirt1 research focused on its role in prolonging the life span of lower organisms, but recent studies have shown that Sirt1 activity can affect a variety of proteins involved in metabolic diseases [27]. Cellular studies have shown that Sirt1 can activate fatty acid oxidation, thereby regulating fat metabolism [28]. Furthermore, specific activation of Sirt1 by SRT1720 protected mice from diet‐induced obesity and insulin resistance [29]. Sirt1‐overexpression in mice reduced the susceptibility to HFD‐induced obesity and hepatic steatosis [30], which is consistent with the results of this study. However, in addition to demonstrating this effect in mice, we also demonstrated that the level of Sirt1 was affected by miR‐22 and negatively correlated with the level of lipids in human hepatocytes.

More than a dozen miRNAs have been found to affect Sirt1 in cancer and cardiovascular diseases, some of which indirectly regulate Sirt1 expression and activity, although most directly act on its mRNA. Examples include miR‐9, miR‐93, miR‐100, miR‐132, miR‐181a/b/c, and miR‐204 [20, 31, 32, 33, 34, 35, 36, 37]. As a tumor suppressor gene, miR‐34a has been found to be downregulated in prostate, colon, and ovarian cancer [38, 39, 40]. Decreased miR‐34a levels alter the metabolism of cancer cells, while inhibition of Sirt1 by the expression of miR‐34a increases acetylated p53 and PUMA, thereby regulating cell cycle progression and apoptosis [41]. Sirt1 also inhibits PPARγ, a key adipogenic transcription factor, while the loss of Sirt1 affects fatty acid beta‐oxidation [42]. Here, we also demonstrated that the overexpression of miR‐22 might lead to changes in the hepatocyte lipid content and weakening of the PPARα signaling pathway through Sirt1.

In addition, Sirt1 has been reported to interfere with insulin secretion by downregulating UCP2 [43], which is important for controlling metabolic homeostasis throughout the body. In adipocytes, overexpression of miR‐132 reduces Sirt1 levels and also promotes the secretion of pro‐inflammatory factors such as IL‐8 and MCP‐1 [31]. In this study, we found that the miR‐22 inhibitors reduced the expression of TNF‐α and IL‐6 in steatotic cells and helped to restore normal cell metabolism.

## Conclusions

In this study, *in vivo* and *in vitro* models of steatosis were used to simulate abnormal lipid deposition in hepatocytes, which can be widely used in other studies. Overexpression and inhibition of miR‐22 was used to verify its effects on lipid metabolism. The results demonstrated that the degree of steatosis was notably aggravated by miR‐22 overexpression. Conversely, steatosis was alleviated after inhibition of miR‐22, indicating that miR‐22 is involved in fat deposition. Furthermore, miR‐22 was validated to have effects on downstream lipid metabolism‐related genes, revealing the pathways through which miR‐22 regulates lipid metabolism. Therefore, miR‐22 may be a promising target for the treatment of NAFLD and other diseases related to abnormal fat metabolism. This study explored and validated the role of miR‐22 in the process of fat deposition, and preliminarily explored the mechanism of miR‐22 regulates fat deposition.

## Conflict of interest

The authors declare no conflict of interest.

## Author contributions

ZY designed the study. ZY, WQ, JW, and LW performed the experiments. ZY analyzed and interpreted data. ZY wrote, reviewed, and revised the manuscript. JH and QZ provided administrative, technical, and material support.

## Data Availability

All data presented in this study are available from the corresponding author upon reasonable request.
